# The tide of dietary risks for noncommunicable diseases in Pacific Islands: an analysis of population NCD surveys

**DOI:** 10.1186/s12889-022-13808-3

**Published:** 2022-08-10

**Authors:** Erica Reeve, Prabhat Lamichhane, Briar McKenzie, Gade Waqa, Jacqui Webster, Wendy Snowdon, Colin Bell

**Affiliations:** 1 Global Obesity Centre, Institute for Health Transformation, School of Health and Social Development, Deakin University, 1 Gheringhap Street, Geelong, VIC 3220 Australia; 2 School of Medicine, Faculty of Health, Deakin University, 75 Pigdons Rd, Waurn Ponds, VIC 3216 Australia; 3 Food Policy Division, The George Institute for Global Health, UNSW, 1 King St, Newtown, Sydney, Australia; 4 Pacific Research Centre for Prevention of Obesity and Non-Communicable Disease (C-POND), Fiji National University, Suva, Fiji

**Keywords:** Pacific Islands, Dietary risk, Noncommunicable diseases, Adults, Change over time

## Abstract

**Objective:**

To describe changes over time in dietary risk factor prevalence and non-communicable disease in Pacific Island Countries (PICTs).

**Methods:**

Secondary analysis of data from 21,433 adults aged 25–69, who participated in nationally representative World Health Organization STEPs surveys in 8 Pacific Island Countries and Territories between 2002 and 2019. Outcomes of interest were changes in consumption of fruit and vegetables, hypertension, overweight and obesity, and hypercholesterolaemia over time. Also, salt intake and sugar sweetened beverage consumption for those countries that measured these.

**Results:**

Over time, the proportion of adults consuming less than five serves of fruit and vegetables per day decreased in five countries, notably Tonga. From the most recent surveys, average daily intake of sugary drinks was high in Kiribati (3.7 serves), Nauru (4.1) and Tokelau (4.0) and low in the Solomon Islands (0.4). Average daily salt intake was twice that recommended by WHO in Tokelau (10.1 g) and Wallis and Futuna (10.2 g). Prevalence of overweight/obesity did not change over time in most countries but increased in Fiji and Tokelau. Hypertension prevalence increased in 6 of 8 countries. The prevalence of hypercholesterolaemia decreased in the Cook Islands and Kiribati and increased in the Solomon Islands and Tokelau.

**Conclusions:**

While some Pacific countries experienced reductions in diet related NCD risk factors over time, most did not. Most Pacific adults (88%) do not consume enough fruit and vegetables, 82% live with overweight or obesity, 33% live with hypertension and 40% live with hypercholesterolaemia. Population-wide approaches to promote fruit and vegetable consumption and reduce sugar, salt and fat intake need strengthening.

**Supplementary Information:**

The online version contains supplementary material available at 10.1186/s12889-022-13808-3.

## Background

Noncommunicable diseases (NCDs), including cardiovascular disease, diabetes, cancer and respiratory disease account for over 70% of worldwide mortality [1]. The majority of this mortality burden (80%) is borne by low and middle-income countries (LMICs) [2–4], where NCDs have a substantial impact on individuals, households and health care systems [5, 6]. Additionally, around 48% NCD deaths in LMICs are considered premature, affecting people under the age of 70 years [7, 8]. The disproportional impact of NCDs on the ‘working-age’ population in LMICs compromises productivity, economic growth and development [9, 10]. Addressing NCDs through improved prevention and treatment has been recognised as a key target in the Sustainable Development Goals.

NCDs and their risk factors are the result of a complex interplay between genes, behaviors and environment [11]. Overweight and obesity, linked primarily to an overconsumption of dietary energy, is strongly associated with an increased prevalence of diabetes, hypertension and cardiovascular disease, as well as increased NCD-related mortality [12]. Food and diet are particularly strong determinants of NCDs including type 2 diabetes [12, 13], cardiovascular disease [14, 15] and a number of cancers [12, 15]. Dietary factors with the strongest correlation to mortality include high sodium intake, low intake of whole grains and low intakes of fruit and vegetables [16–18]. Diets that are high in sugar [19] and fat (particularly trans-fats and saturated fats) [20] also increase NCD risk. Collectively, dietary risks are the second leading risk factor attributable to global mortality for females, and the third leading risk for males [13].

Dietary risk factors in particular are of concern in Pacific Island countries, where nearly 3 of every 4 deaths are due to NCDs [21]. Pacific Island countries comprise 9 out of 10 of the most obese nations in the world, and a diabetes prevalence of 40% in adults is common among Pacific countries [22]. Studies have demonstrated a correlation between metabolic syndrome and NCDs including diabetes, cancer and cardiovascular diseases, and substantial dietary transition occurring in Pacific Island countries in recent years [23–26]. The dietary transition involved a displacement of diets traditionally high in fruit and vegetables and other fresh produce high in fibre, vitamins and low dietary sodium and fat [24, 27–29] with processed foods high in sodium, hydrogenated fat and sugar, including edible oils, sauces and condiments, noodles, baked goods and processed meats [23, 24, 26, 27, 29]. These changes were triggered by multiple factors, including socioeconomic changes and increasing participation in globalised food systems [30]. The dietary transition has seen a 40% increase in processed food sales in Pacific countries between 2004 and 2018 [29].

Concerned about the impact of these changes on individual and community health as well as national economies [32], Pacific governments have introduced a range of population-wide initiatives for preventing diet related NCDs [33–35]. Pacific countries have implemented taxes on SSB [31] and/or policies to reduce sales and marketing of unhealthy food in schools [32, 33]. Tonga, Samoa and Fiji have used import excises to reduce sales of unhealthy fats and oils [34, 35] or fatty meat cuts [36, 37]. Regionally, countries report against a framework for monitoring NCD prevention actions [38, 39]. A stabilisation of diet-related NCD risk factors would be a promising sign preventive efforts are working.

However, there is a dearth of dietary intake data in the Pacific Islands [40], and the high cost of conducting national food surveys [43], together with the limited capacity for data collection and analysis [41, 42], have made it difficult to examine the impact of policy on diet and NCDs. Also, few studies have examined changes in risk factor prevalence over time [43].

In this paper we examine how diet-related NCD risk factors have changed in 8 Pacific countries that have completed two WHO STEPs (STEPwise approach to surveillance) surveys [46].

## Methods

### Data source

STEPs surveys apply standardized and internationally recognized methods to collect data on a range of NCD risk factors including dietary behaviors (including sodium, sugar, fruit and vegetable intake), risk factors (hypertension, hypercholesterolemia, overweight and obesity) and health outcomes (diabetes) [44]. Since 2002, STEPs have been conducted in countries across the Pacific every 5 to 10 years. We conducted a secondary analysis using summary data from STEPS reports. The survey targets a representative sample of adults aged between 18 and 69 years and gathers data via questionnaires, physical measurements and biochemical measurements. It has been designed so that each county measures a core set of risk factors using standardized methods so that comparisons can be made over time within a country and between countries. Countries have the option of adding modules on additional risk factors or questions that capture more information on the core set of risk factors [43]. Full detail on STEPS survey methodology is described elsewhere [44]. Published STEPS reports were accessed online from WHO and/or governments websites. At the time of this analysis, no Pacific country had published more than two STEPs reports. Because we sourced publicly available data ethics approval was not sought.

### Data extraction

We extracted data on modifiable dietary risk factors (fruit and vegetable intake) and specific dietary conditions (overweight and obesity, hypertension, hypercholesterolemia) that were collected in a similar manner across two time points. Most recent surveys in PICTs have added behavioral questions on intakes of sugar or sodium. Because of a growing awareness of the NCD risk associated with sugar and salt in PICTs [31, 45, 46] and focus in food policy [47, 48] we also report sugar sweetened beverage (SSB) consumption and sodium intake where they were measured in the second round (these were largely absent from the first round). Data was extracted into an excel form by two different authors. Because we were interested in risk profiles by sex, data were disaggregated by sex and age strata, usually capturing samples between 25 and 64 years of age in 5 years, 10 years or twenty-year groups. We elected not to extract data on hyperglycemia given issues with blood glucose measurement in some STEPs surveys [49]. Table 1 provides definitions for the extracted risk factors and conditions.

**Table 1:** Risk factor definitions

Risk factor/Condition	Definition
Fruit and vegetable consumption	Proportion of participants consuming less than 5 servings of fruits & vegetables per day
Sugar-sweetened beverage consumption	Mean number of servings of sugary drinks consumed per day (defined as one can or one large glass of fizzy drink, squash, cordial, drink concentrates and juice drinks, excluding pure unsweetened fruit juice).
Added salt	Proportion of people who reported always or often added salt or to food before or while eating
Salt intake	Mean salt intake (g/day) based urinary sodium and creatinine
Overweight and obesity	Proportion of participants living with overweight or obesity (BMI greater than or equal to 25)
Hypertension	Proportion of people with SBP > 140 and/or DBP > 90 mmHg and/or currently on medication for raised BP
Hypercholesterolemia	Number of participants with raised total cholesterol (≥5.2 mmol/L or ≥ 200 mg/dl)

### Data analysis and reporting

We employed a direct standardization technique to calculate age standardized rates for each countries in preference to using crude age specific rates could be misleading because of the differences in underlying composition of the populations. The WHO standard population grouped in 5-year intervals [50] was used to calculate age-standardized rates for each indicator using *dstdize* command in Stata v17.0 [51]. A 95% confidence interval was calculated using the methods described by Breslow and Day [52]. For Tokelau, the confidence interval was not calculated as the whole target population was included in the survey. Data was only from the STEPs surveys in bands of 20 years or greater than 20 years (45–64 years / 45–69 years) and the Cook Islands and Wallis and Futuna used a non-standard age group band of 18–44 years in the second-round surveys. Hence, unstandardized rates have been presented for these countries along with confidence intervals that have been computed using exact binomial method.

We present data for individual countries and pooled prevalence between survey periods to give an indication of overall changes in risk factor prevalence for these 8 countries. The age-standardised rates were pooled using *metaprop* command to calculate the pooled prevalence using a fixed effect model [53]. The pooled weighted estimate was calculated using the inverse variance method after Freeman-Tukey Double Arcsine Transformation to stabilize the variances [53]. Exact binomial confidence interval was calculated for each pooled estimate. Test of proportion was conducted to examine the statistical difference between two rounds of surveys.

## Results

Eight countries, Cook Islands, Fiji, Kiribati, Nauru, Solomon Islands, Tokelau, Tonga and Wallis and Futuna, have two published NCD survey reports giving us an overall sample of 12,076 for first round survey and 9357 for second round survey (Tables 2 and 3). The time between surveys in each country ranged from 8 to 11 years (mean = 9.75 years).

**Table 2:** Number of participants with information on dietary NCD risk factors in survey round one

Country	Survey 1	Age range	Five fruit and veg	Overweight and obesity	Hypertension	Hypercholesterol
Cook Islands	2003	45–64	985	939	950	871
Fiji	2002	25–64	NA	4190	5012	NA
Kiribati	2004	25–64	1329	1351	1368	741
Nauru	2004	25–64	1653	1710	1705	1726
Solomon Islands	2005	25–69	1910	1665	1702	470
Tokelau	2006	25–64	392	427	333	427
Tonga	2004	24–64	848	844	848	847
Wallis and Futuna	2009	45–64	146	162	158	NA
Total			7263	11,279	12,076	5236

**Table 3:** Number of participants with information on dietary NCD risk factors in survey round two, and time lapsed between surveys

Country	Survey 2	Approximate timeframe since Survey 1 (years)	Age range	Five fruit and veg	SSB Daily	Overweight and obesity	Hypertension	Hyperglycemia	Hypercholesterol
Cook Islands	2013	10	45–64	611	NA	430	411	346	368
Fiji	2011	9	25–64	NA	NA	2526	2548	2378	NA
Kiribati	2015	11	30–69	1329	1900	1351	1368	861	741
Nauru	2015	11	25–64	838	1317	861	691	667	668
Solomon Islands	2015	10	30–69	1856	2443	1440	1472	1340	1342
Tokelau	2015	9	30–69	390	547	384	387	382	288
Tonga	2012	8	25–69	2438	NA	2273	2332	2287	2065
Wallis and Futuna	2019	10	45–64	661	NA	626	628	606	NA
Total				8123	6207	9195	8187	8851	9195

### Fruit and vegetable consumption

**Fig. 1:**
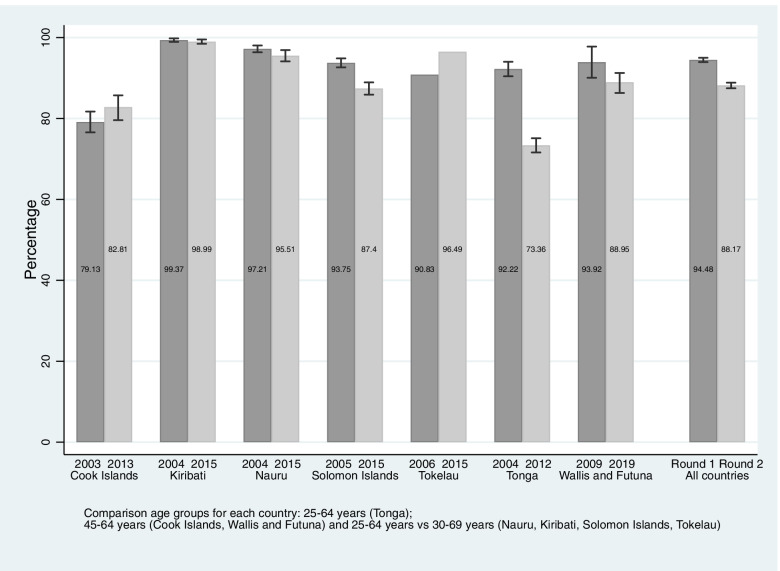
Age-standardized prevalence of adults aged 25–69 years consuming less than five servings of fruits and vegetables per day by survey year and country

Figure 1 reports age-standardized prevalence of adults consuming less than 5 serves of fruits and vegetables per day. Prevalence decreased significantly in Tonga from 92.2% (95%CI: 90.4, 94.0) to 73.4% (95%CI: 71.6, 75.1) over 8 years, and in the Solomon Islands from 93.8% (95%CI: 92.6, 94.9) to 87.4% (95%CI: 85.9, 88.9) over 9 years. In both countries statistically significant reductions were observed for both women and men (see Supplementary File 1). In Nauru and Wallis and Futuna, prevalence decreased statistically significantly for men only, from 98.4% (95%CI: 97.6, 99.4) to 94.84% (95%CI: 92.5, 97.2) and 96.3% (95%CI: 92.3, 100.3) to 88.3 (95%CI: 83.9, 91.8) respectively. In Tokelau on the other hand, prevalence increased from 90.8 to 96.5% over the 9 years between 2006 and 2015.

The pooled analysis revealed a significant decrease in the proportion of adults consuming less than 5 serves of fruit and vegetables per day, from 94% (95%CI: 93.9, 94.5) to 88% (95%CI: 87.5, 88.2), significant for both men and women.

### Sugary drink consumption

Four of the countries measured sugary drink consumption in Survey 2. Adults in Kiribati, Nauru and Tokelau (across both sexes) reported consuming over 3.5 sugary drinks each per day. In contrast, Solomon Islands adults reported consuming an average of 0.4 sugary drinks per day. SSB consumption did not vary significantly between men and women (Table 4).

**Table 4:** Average daily consumption of sugary drinks by adults in Survey 2

Country (survey year)	Age group	Average serves^a^ per day (95%CI)		
		Men (%)	Women (%)	Both (%)
Kiribati (2015)	18–69	3.5 (1.6–5.4)	3.9 (1.9–5.8)	**3.7** (2.0–5.5)
Nauru (2015)	18–69	3.9 (3.4–4.4)	4.3 (3.4–5.2)	**4.1** (3.6–4.6)
Solomon Islands (2015)	18–69	0.3 (0.3–0.4)	0.3 (0.2–0.4)	**0.4** (0.3–0.5)
Tokelau^b^ (2014)	18–69	3.9	4.1	**4.0**

^a^A sugary drink is defined as fizzy drink, squash, cordial, drink concentrates and juice drinks excluding pure unsweetened fruit juice. One serving is defined as one can of drink, or one large glass

^b^No CI as entire target population was included in the survey

### Adding salt to meals before consumption

Mechanisms for measuring salt varies significant across the included surveys. Five countries asked about ‘always or often’ adding salt before eating or when eating (Cooks, Kiribati, Tokelau, Solomon Islands, Nauru) (Table 5). Nauru and Cook Islands reported the per cent of participants ‘always or often’ eating processed food high in salt, and applied a likert scale querying participants on the importance of lowering dietary salt. Because of this variation we only extracted data on the percent of adults in Survey 2 ‘always or often’ adding salt to meals before eating. The proportion of adults ‘always or often’ adding salt to meals before eating ranged from 31.6% in Tokelau (higher for women than men) to 65.4% (60.5–70.3) in Nauru. Based on urinary analysis, adults in Tokelau, consumed an average of 10.1 g/day of salt, and consumption was higher for men (12.0 g/day) than women (8.6 g/day). In Wallis and Futuna salt consumption was 10.2 g/day, also higher for men (11.7 g/day) than women (8.8 g/day).

**Table 5:** Percent of adults ‘always or often’ adding salt before eating

Survey	Age group (years)	Adults who add salt ‘always or often’ before eating or when eating (95%CI)^a^		
		Men (%)	Women (%)	Both (%)
Cook Islands	18–64	37.3 (33.9–40.7)	35.7 (32.9–38.5)	**36.4** (34.3–38.6)
Kiribati	18–69	34.5 (27.6–41.4)	47.0 (37.4–56.6)	**41.3** (33.7–48.9)
Nauru	18–69	63.5 (60.5–66.4)	67.1 (60.1–74.2)	**65.4** (60.5–70.3)
Solomon Islands (2015)	18–69	48.8 (43.0–54.7)	44.6 (39.9–49.2)	**46.6** (42.0–51.1)
Tokelau (2014)^b^	18–69	25.8	36.6	**31.6**
		Average salt intake based on urinary sodium (g/day)		
Tokelau (2014)^b^	18–69	12.0	8.6	**10.1**
Wallis and Futuna (2019)	18–69	11.7 (11.5–12.0)	8.8 (8.7–9.0)	**10.2 (9.8–10.5)**

^a^Dietary salt includes ordinary table salt, unrefined salt such as sea salt, iodized salt, salty stock cubes and powders, and salty sauces such as soya sauce or fish sauce. This question relates to salt added directly before consumption (regardless of meal composition)

^b^No CI due to measuring entire population

### Overweight and obesity

**Fig. 2:**
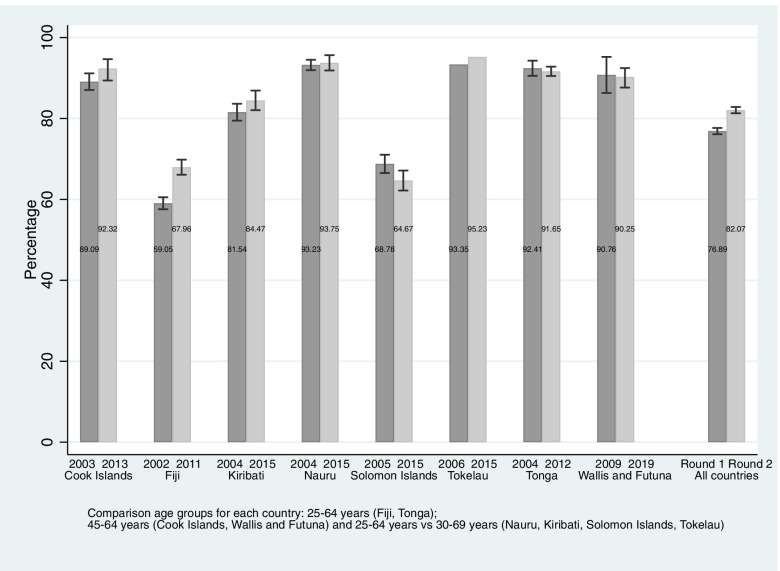
Age-standardized prevalence of adults aged 25–69 years living with overweight and obesity

Figure 2 reports age-standardized prevalence of adults living with overweight and obesity. There was a statistically significant increase in prevalence from 59.1% (95%CI: 57.5, 60.5) to 67.96% (95%CI: 66.1, 69.8) in Fiji largely attributable to an increase for women from 75.2% (95%CI: 74.1, 76.3) to 85.3% (95%CI: 84.4, 86.3). Prevalence also increased in Tokelau from 93.3% to 95.2%, particularly for women (94.5% to 95.4%). Women lived with a higher prevalence of overweight and obesity than men in all countries except Nauru. No significant changes in prevalence were observed for the Cook Islands, Kiribati, the Solomon Islands or Tonga. The pooled analysis revealed a significant increase from 76.9% (95%CI: 76.1, 77.7) to 82.1% (95%CI: 81.3, 82.9) in the proportion of adults living with overweight or obesity.

### Adults living with hypertension

**Fig. 3:**
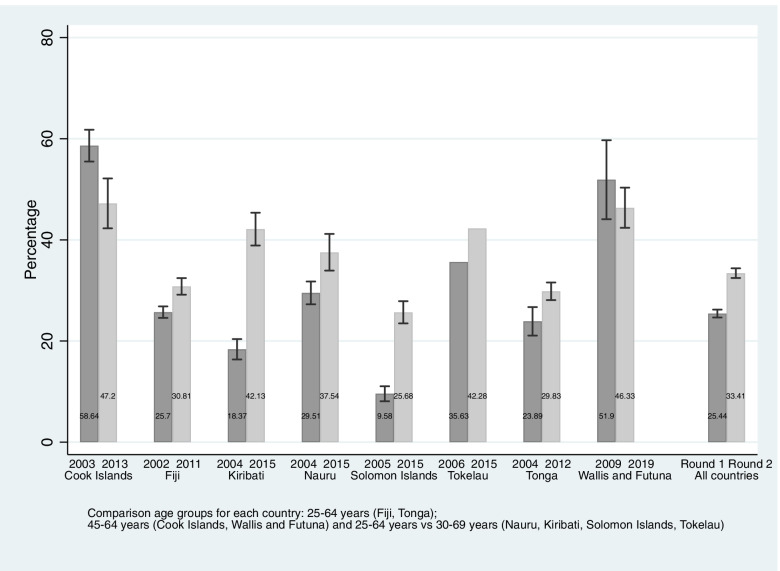
Age-standardized prevalence of adults aged 25–69 years living with hypertension by survey year and country

Prevalence of hypertension increased in 6 countries (Fig. 3). In Kiribati prevalence increased from 18.4% (95%CI: 16.4, 20.4) to 42.13% (95%CI: 38.9, 45.4), in the Solomon Islands from 9.6% (95%CI: 8.1, 11.1) to 26.83% (95%CI: 23.5, 27.9), in Nauru from 29.5% (95%CI: 27.3, 31.8) to 37.6% (95%CI: 33.9, 41.2)], in Tokelau from 35.6% to 42.4%), in Tonga from 23.9% (95%CI: 21.1, 26.7) to 29.8% (95%CI: 28.1, 31.6) and in Fiji from 25.7% (95%CI: 24.6, 26.8) to 30.81% (95%CI: 29.2, 32.5) (Fig. 3). Increases were significant for women in all countries and for men except in Nauru and Tonga. Against this pattern, hypertension prevalence decreased from 58.6 (95%CI: 55.5, 61.8) to 47.2 (95%CI: 42.3, 52.2) in the Cook Islands driven by a large decrease for men.

The pooled analysis showed an overall increase in the prevalence of hypertension from 25.4% (95%CI: 24.7, 26.2) to 33.41% (95%CI: 32.5, 34.4) across the 8 countries.

### Adults living with hypercholesterolemia

**Fig. 4:**
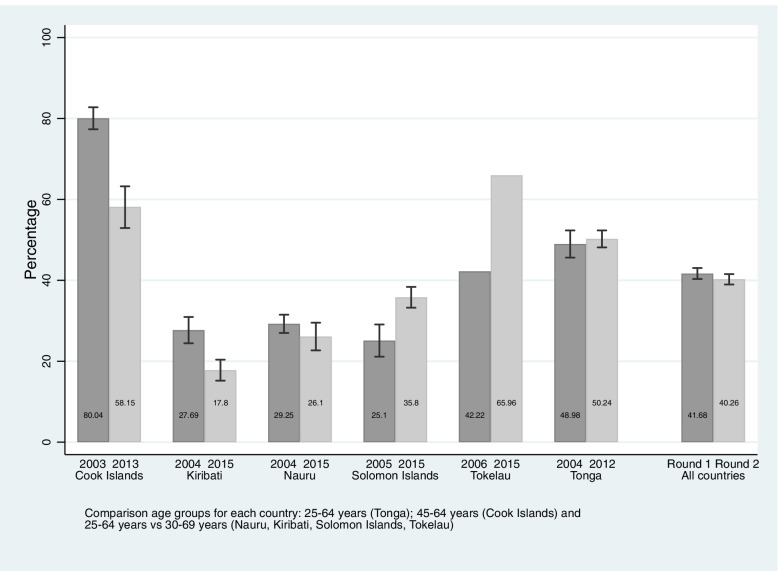
Age-standardized prevalence of adults aged 25–69 years living with raised total cholesterol by survey year and country

Six countries had comparable measures for hypercholesterolaemia (Fig. 4). Prevalence increased from 25.1% (95%CI: 21.1, 29.1) to 35.8% (33.2, 38.4) in the Solomon Islands and from 42.2% to 65.96% in Tokelau. Prevalence decreased from 80.0% (95%CI: 77.3, 82.8) to 58.2% (95%CI: 63.2, 52.9) in the Cook Islands, and from 27.7% (95%CI: 24.4, 30.9) to 17.8% (95%CI: 20.4, 15.2) in Kiribati. Significant reductions were observed for men and women in both countries.

## Discussion

We used nationally representative survey data from 8 Pacific Island Countries and Territories to assess changes over time in dietary risk factor prevalence. Some reductions in risk were observed, including statistically significant reductions in the proportion and adults consuming < 5 servings of fruit and vegetables per day. However, the prevalence of those living with overweight or obesity increased significantly in Fiji and Tokelau as did hypertension in 6 countries and hypercholesterolaemia in the Solomon Islands and Tokelau. Salt consumption was twice the 5 g per day recommendation of WHO in the two countries that conducted urinary analysis, and adults in Kiribati, Nauru and Tokelau were consuming up to an average of 4 serves per day of SSBs. Most Pacific adults (88%) do not consume enough fruit and vegetables, 82% live with overweight or obesity, 33% live with hypertension and 40% live with hypercholesterolaemia.

### Dietary risk profile in the Pacific Islands

Our results align with literature describing a steady increase in overweight and obesity in Pacific Island countries [23, 24, 54]. However, in our study this increase was driven by just two countries, Fiji and Tokelau, and in particular by an increase in prevalence for women in Fiji. Fiji was one of the first Pacific countries to complete a STEPS survey, and the timing of the survey (eariery in the processed food transition) may have contributed to lower baseline prevalence compared to other countries. Our observation of increasing overweight and obesity in women compared to men in Fiji is consistent with other studies in LMICs [55–57]. Gender weight disparities may be a result of sociocultural factors, or because men are more often engaged in highly physical occupations compared with women, and involvement in sports is still less common in women [57]. In 6 of the 8 countries, there was no significant increase in overweight and obesity prevalence and mean BMIs were also relatively stable. This contrasts with other countries, including the US [58], where rates of obesity (BMI > 30) have accelerated faster than rates of overweight (BMI > 25) in recent years. High baseline levels of overweight and obesity in Pacific countries may have contributed to this stabilisation, noting that some Pacific populations have less fat mass at a given BMI than Caucasian populations [59]. It is also possible that preventive measures are starting to make a difference in some countries.

Pacific health and agricultural agencies have proactively promoted fruit and vegetable consumption [60, 61] in recent years, and offered agricultural support programs for farmers [60, 62] which may have contributed to the decrease over time in the proportion of adults consuming < 5 servings of fruit and vegetables each day. Despite this decrease, 88% of Pacific adults still report inadequate consumption. That this is consistent with the global dietary transition away from plant-based diets makes it no less concerning, and it points to the need to strengthen the efforts mentioned above. Inadequate fruit and vegetable consumption is an important but often neglected risk factor for NCDs [63], and a challenge across most regions of the world [64], particularly in LMICs [65]. A study of fruit and vegetable consumption in 28 LMICs between 2005 and 16 found that only 18% (16.6–19.4%) of adults over 15 years consumed WHO recommended amounts [65]. Consumption increased with GDP and secondary education but decreased with food pricing instability. Fiji and Tonga both relaxed import duties on fruit and vegetables, although evidence from Tonga suggest that this may have only benefited traders [66]. These findings point to the need to strengthen food systems approaches that promote production of resilient, biodiverse crops, and address post-harvest losses and market access [67].

An emerging concern for Pacific countries is high SSB consumption [68]. Adults in Nauru, Tokelau, and Kiribati consumed more than 3.5 serves of sugary beverages per day. Similarly high average daily serves have been observed in Tuvalu (3 serves/day) based on their STEPS survey. A recent study of trade data from 12 Pacific Island countries documented a 65% increase to sugary drink imports between 2000 and 2015 [69]. In this study, the Solomon Islands stood out from other countries with adults reporting consuming 0.3 average daily serves of SSBs. This may be attributable to the Solomon Islands being at an earlier stage of the global dietary transition than other Pacific countries, remoteness from markets, or the high volume of sweetened tea/coffee beverage powders consumed [70], which may not have been adequately captured by STEPS. Many Pacific Islands countries having adopted taxes on SSBs [31], but these may need to be increased in order to make meaningful shifts to consumption, and the sale of SSBs in and around educational institutions could be tightened [71, 72]. The Solomon Islands in particular may benefit from introducing an SSB tax to keep consumption levels low [70].

The average prevalence of hypertension increased from a quarter to a third of all adults in these Pacific countries, with prevalence levels similar to Australia (34%) [73], possibly due to the high un-met needs in controlling blood pressure in Pacific countries [74, 75]. While further surveys are needed to confirm a trend, persistently high (and potentially increasing) rates of hypertension signal a future pipeline of vascular diseases with a potentially overwhelming impact on Pacific health systems and economies [76]. Dietary sodium, saturated fats and trans fats are major dietary contributors to hypertension [75] and saturated and trans fat are major dietary contributors to hypercholesterolemia [77, 78]. In this study, overall prevalence of hypercholesterolemia was over 40%, and in the two countries where salt intake was measured, it was over 10 g/day, more than double that recommended by WHO. These indicators support the need to disrupt current dietary patterns in the Pacific, specifically excessive consumption of fatty meat, hydrogenated vegetable oil [26, 29, 79], and foods high in sodium [28, 80].

### Policy response to dietary NCD risk factors

Our analysis highlights the ongoing challenge that Pacific countries face in responding to dietary causes of NCDs. Unhealthy dietary patterns are fueled by increased trade liberalization [81–83], the penetration of food marketing [84, 85], and by food environments that promote affordable and convenient processed foods that are high in energy, salt, sugar and fat [27]. Additionally, policymakers have faced strong opposition from food and beverage companies trying to diminish policies [86], and pressures to minimize impacts of food policy on trade participation [37]. The multisectoral nature of food policy has made it difficult for Pacific leaders to implement and then enforce all recommended policy measures [87, 88], leading many to favour ‘softer’ approaches (i.e. guidelines and promotional materials) over regulatory approaches. Further, Pacific Island countries have struggled to find capacity to carry out regular dietary surveys and demonstrate the potentially positive impact of food environment policies on consumption [40]. Pacific Island MANA as a component of Framework of action for revitalization of healthy islands in the Pacific has been an important step to promoting political accountability to NCD prevention [38, 39], but countries will need to adopt a stronger cross sectoral approaches towards regulating, monitoring and enforcing food environment policies [33, 39, 89].

### Strengthening surveillance of NCD risk factors

The purpose of STEPS is to provide a standardized method for collecting, analysing and disseminating data on key NCD risk. In the Pacific, STEPs surveys are used to inform high-level economic discussions [90], for regional monitoring and accountability strategies [39], to contribute to global monitoring, and to underpin evidence-based policymaking at the national level [70, 91]. By gathering STEPs data from multiple countries and over two time points we identified several opportunities to strengthen NCD risk factor monitoring in Pacific countries. Firstly, standardizing age grouping between survey rounds and countries would aid interpretation of published survey reports. For instance, Nauru, Kiribati and Solomon Islands reported results in the groupings of 25–34, 35–44, 45–54 and 55–64 years in the first round while the groupings were 18–29, 30–44 and 45–69 years in the second round. Secondly, standardizing risk factor thresholds or cut points between countries and survey rounds. In Fiji for instance, 2002 fruit and vegetable consumption was reported as the percent of people reporting < 1 serve of fruit and vegetable per day, whereas in 2011, it was the percent consuming < 5 serves. Thirdly, standardized time intervals between the surveys. Fourthly reducing the lag between data collection and publication of study reports so timely action can be taken. Finally salt intake, in particular, needs to be reported consistently, perhaps in place of less useful measures such as self-reported oil intake [92].

### Strengths and limitations

#### Strengths

There are several strengths of this study. To our knowledge, this is the first paper comparing shifts in dietary risk factors over time in multiple Pacific countries. We used standardized rates rather than crude rates to make this comparison. Also, this data makes use of the reports generated by the Pacific countries for guiding and evaluating prevention efforts. We used standardized rates rather than crude rates to make this comparison. Additionally, by pooling prevalence, this paper shed light on NCD risk factor prevalence at a semi-regional level, providing critical information to guide the efforts of regional agencies, and those interested in dietary patterns of NCD risk in LMICs more broadly.

#### Limitations

There were limitations to the approach taken in our study, in addition to those raised above as opportunities to strengthen NCD surveillance [93]. This was a secondary analysis dependent on data summaries in published reports rather than raw data.

We did not report other NCD risk factors such as physical activity levels or tobacco and alcohol use. We were not able to report on hyperglycemia, which is a key risk factor in this Region, due to errors in that date reported previously [36]. Further, we did not present data from United States affiliated Pacific countries as many of these countries use an alternative NCD surveillance system to STEPs. Finally, two time points provide limited insight on change over time.

## Conclusions

While some of the eight Pacific countries included in this analysis experienced reductions in diet-related NCD risk factors over time, most did not. Most Pacific adults (88%) do not consume enough fruit and vegetables, 82% live with overweight or obesity, 33% live with hypertension and 40% live with hypercholesterolaemia. Population-wide approaches to promote fruit and vegetable consumption and reduce sugar, salt and fat intake need strengthening. The value of STEPS surveys for monitoring trends in NCD risk will be fully realized when countries have conducted at least three surveys, though this requires a more consistent measurement of risk factors over time.

## Supplementary Information

**Additional file 1.**

## Data Availability

This study was based on publicly available published survey reports, and the compiled dataset can be made available from the corresponding author on reasonable request.

## Abbreviations

BMI: Body Mass Index
CI: Confidence Interval
LMIC: Low and middle-income countries
NCD: Noncommunicable diseases
STEPs: STEPwise approach to surveillance

## References

[1] Bennett JE, Stevens GA, Mathers CD, Bonita R, Rehm J, Kruk ME, et al. NCD countdown 2030: worldwide trends in non-communicable disease mortality and progress towards sustainable development goal target 3.4. Lancet. 2018;392(10152):1072–88. 10.1016/S0140-6736(18)31992-5 30264707

[2] Benziger CP, Roth GA, Moran AE (2016). The global burden of disease study and the preventable burden of NCD. Glob Heart.

[3] World Health Organization (2018). Noncommunicable diseases: key facts.

[4] Nugent R, Bertram MY, Jan S, Niessen LW, Sassi F, Jamison DT (2018). Investing in non-communicable disease prevention and management to advance the sustainable development goals. Lancet.

[5] Murphy A, Palafox B, Walli-Attaei M, Powell-Jackson T, Rangarajan S, Alhabib KF (2020). The household economic burden of non-communicable diseases in 18 countries. BMJ Glob Health.

[6] Lee JT, Hamid F, Pati S, Atun R, Millett C (2015). Impact of noncommunicable disease multimorbidity on healthcare utilisation and out-of-pocket expenditures in middle-income countries: cross sectional analysis. Plos One.

[7] World Health Organization (2014). Global status report on noncommunicable diseases.

[8] Wou C, Unwin N, Huang Y, Roglic G (2019). Implications of the growing burden of diabetes for premature cardiovascular disease mortality and the attainment of the sustainable development goal target 3.4. Cardiovasc Diagn Ther.

[9] Orueta JF, Nugent RA, Husain MJ, Kostova D, Chaloupka F. Introducing the PLOS special collection of economic cases for NCD prevention and control: a global perspective. Plos One. 2020;15(2). 10.1371/journal.pone.0228564 PMC7004318 32027710

[10] Nikolic I, Stanciole AE, Zaydman M (2011). Chronic emergency: why NCDs matter.

[11] World Health Organization. Noncommunicable diseases: Newsroom factsheet: World Health Organization. [cited 2022] Available from: https://www.who.int/news-room/fact-sheets/detail/noncommunicable-diseases.

[12] Global Burden of Disease Obesity Collaborators (2017). Health effects of overweight and obesity in 195 countries over 25 years. N Engl J Med.

[13] Murray CJL, Aravkin AY, Zheng P, Abbafati C, Abbas KM, Abbasi-Kangevari M (2020). Global burden of 87 risk factors in 204 countries and territories, 1990–2019: a systematic analysis for the global burden of disease study 2019. Lancet.

[14] Anand SS, Hawkes C, de Souza RJ, Mente A, Dehghan M, Nugent R (2015). Food consumption and its impact on cardiovascular disease: importance of solutions focused on the globalized food system: a report from the workshop convened by the world heart federation. J Am Coll Cardiol.

[15] Australian Institute of Health and Welfare (2017). Impact of overweight and obesity as a risk factor for chronic conditions.

[16] Afshin A, Sur PJ, Fay KA, Cornaby L, Ferrara G, Salama JS (2019). Health effects of dietary risks in 195 countries, 1990–2017: a systematic analysis for the global burden of disease study 2017. Lancet.

[17] Wang X, Ouyang Y, Liu J, Zhu M, Zhao G, Bao W (2014). Fruit and vegetable consumption and mortality from all causes, cardiovascular disease, and cancer: systematic review and dose-response meta-analysis of prospective cohort studies. BMJ.

[18] Nguyen B, Bauman A, Gale J, Banks E, Kritharides L, Ding D. Fruit and vegetable consumption and all-cause mortality: evidence from a large Australian cohort study. Int J Behav Nutr Phys Act. 2016;13:9. 10.1186/s12966-016-0334-5 PMC4727264 26810760

[19] World Health Organization (2015). Guideline: sugars intake for adults and children.

[20] Sacks FM, Lichtenstein AH, Wu JHY, Appel LJ, Creager MA, Kris-Etherton PM (2017). Dietary fats and cardiovascular disease: a presidential advisory from the American Heart Association. Circulation.

[21] World Health Organization (2019). Factsheet: Noncommunicable diseases in the Western Pacific.

[22] Win Tin ST, Lee CMY, Colagiuri R (2015). A profile of diabetes in Pacific Island countries and territories. Diabetes Res Clin Pract.

[23] Hughes RG, Marks GC (2009). Against the tide of change: diet and health in the Pacific islands. J Am Diet Assoc.

[24] Hughes RG. Diet, food supply and obesity in the Pacific: World Health Organization; 2003.

[25] Coyne T (2000). Lifestyle diseases in Pacific communities. Secretariat of the Pacific Community.

[26] DiBello JR, McGarvey ST, Kraft P, Goldberg R, Campos H, Quested C, et al. Dietary patterns are associated with metabolic syndrome in adult Samoans. J Nutr Nutrition Epidemiol. 2009. 10.3945/jn.109.107888 PMC2744614 19710163

[27] Seiden A, Hawley NL, Schulz D, Raifman S, McGarvey ST (2012). Long-term trends in food availability, food prices, and obesity in Samoa. Am J Hum Biol.

[28] Snowdon W, Raj A, Reeve E, Guerrero RL, Fesaitu J, Cateine K, et al. Processed foods available in the Pacific Islands. Glob Health. 2013;9(53). 10.1186/1744-8603-9-53 PMC4016479 24160249

[29] Sievert K, Lawrence M, Naika A, Baker P. Processed foods and nutrition transition in the Pacific: regional trends, patterns and food system drivers. Nutrients. 2019;11(6). 10.3390/nu11061328 PMC6628317 31200513

[30] Sahal Estime M, Lutz B, Strobel F (2014). Trade as a structural driver of dietary risk factors for noncommunicable diseases in the Pacific: an analysis of household income and expenditure survey data. Glob Health.

[31] Teng A, Snowdon W, Win Tin ST, Genc M, Na'ati E, Puloka V, et al. Progress in the Pacific on sugar-sweetened beverage taxes: a systematic review of policy changes from 2000 to 2019. Aust N Z J Public Health. 2021;45(4):376–84. 10.1111/1753-6405.13123 34097355

[32] World Health Organization. Meeting Report: Regional Workshop on Regulating the Marketing and Sale of Foods and Non-alcoholic Beverages at Schools. Manila, Philippines: 2016.

[33] Reeve E, Thow AM, Bell C, Soti-Ulberg C, Sacks G. Identifying opportunities to strengthen school food environments in the Pacific: a case study in Samoa. BMC Public Health. 2021;21(1):246. 10.1186/s12889-021-10203-2 PMC7844953 33514338

[34] Bell C, Latu C, Na'ati E, Snowdon W, Moodie M, Waqa G. Barriers and facilitators to the introduction of import duties designed to prevent noncommunicable disease in Tonga: a case study. Global Health. 2021;17(1):136. 10.1186/s12992-021-00788-z PMC8626938 34838081

[35] Coriakula J, Moodie M, Waqa G, Latu C, Snowdon W, Bell C. The development and implementation of a new import duty on palm oil to reduce non-communicable disease in Fiji. Global Health. 2018;14(1):91. 10.1186/s12992-018-0407-0 PMC6116374 30157872

[36] Thow AM, Reeve E, Naseri T, Martyn T, Bollars C. Food supply, nutrition and trade policy: reversal of an import ban on turkey tails. Bull World Health Organ. 2017;95(10):723–5. 10.2471/BLT.17.192468 PMC5689196 29147046

[37] Thow AM, Swinburn B, Colagiuri S, Diligolevu M, Quested C, Vivili P, et al. Trade and food policy: Case studies from three Pacific Island countries. Food Policy. 2010;35(6):556–64.

[38] Tolley H, Snowdon W, Wate J, Durand AM, Vivili P, McCool J, et al. Monitoring and accountability for the Pacific response to the non-communicable diseases crisis. BMC Public Health. 2016;16:958. 10.1186/s12889-016-3614-8 PMC5018177 27613495

[39] Win Tin ST, Kubuabola I, Ravuvu A, Snowdon W, Durand AM, Vivili P, et al. Baseline status of policy and legislation actions to address non communicable diseases crisis in the Pacific. BMC Public Health. 2020;20(1):660. 10.1186/s12889-020-08795-2 PMC7216373 32398159

[40] Santos JA, McKenzie B, Trieu K, Farnbach S, Johnson C, Schultz J, et al. Contribution of fat, sugar and salt to diets in the Pacific Islands: a systematic review. Public Health Nutr. 2019;22(10):1858–71. 10.1017/S1368980018003609 PMC6670018 30612591

[41] Lum M, Bennett O, Whittaker M. Strengthening Issues and challenges for health information systems in the Pacific: Findings from the Pacific Health Information Network Meeting 29 September – 2 October 2009 and the Pacific Health Information Systems Development Forum 2–3 November 2009.

[42] Richards NC, Gouda HN, Durham J, Rampatige R, Rodney A, Whittaker M. Disability, noncommunicable disease and health information. Bull World Health Organ. 2016;94(3):230–2. 10.2471/BLT.15.156869 PMC4773929 26966336

[43] Kessaram T, McKenzie J, Girin N, Roth A, Vivili P, Williams G, et al. Noncommunicable diseases and risk factors in adult populations of several Pacific Islands: results from the WHO STEPwise approach to surveillance. Aust N Z J Public Health. 2015;39(4):336-43. 10.1111/1753-6405.12398 PMC4744741 26095921

[44] World Health Organization. NCD surveillance tools: STEPwise Approach to NCD Risk Factor Surveillance (STEPS). Available from: https://www.who.int/teams/noncommunicable-diseases/surveillance/systems-tools/steps.

[45] Aldwell K, Caillaud C, Galy O, Frayon S, Allman-Farinelli M. Tackling the Consumption of High Sugar Products among Children and Adolescents in the Pacific Islands: Implications for Future Research. Healthcare (Basel). 2018;6(3). 10.3390/healthcare6030081 PMC6163880 30002327

[46] Webster J, Waqa G, Thow A-M, Allender S, Lung T, Woodward M, et al. Scaling-up food policies in the Pacific Islands: protocol for policy engagement and mixed methods evaluation of intervention implementation. Nutrition J. 2022;21(1):8. 10.1186/s12937-022-00761-5 PMC8807012 35105346

[47] Reeve E, Naseri T, Martyn T, Bollars C, Thow AM. Developing a context-specific nutrient profiling system for food policy in Samoa. Health Promot Int. 2019;34(6):e94-e105. 10.1093/heapro/day089 30388231

[48] Christoforou A, Snowdon W, Laesango N, Vatucawaqa S, Lamar D, Alam L, et al. Progress on salt reduction in the Pacific Islands: from strategies to action. Heart Lung Circ. 2015;24(5):503-9. 10.1016/j.hlc.2014.11.023 25577701

[49] Taylor R, Zimmet P, Naseri T, Hufanga S, Tukana I, Magliano DJ, et al. Erroneous inflation of diabetes prevalence: Are there global implications? Journal of Diabetes. 2016;8(6):766-9. 10.1111/1753-0407.12447 27400903

[50] Ahmad OB, Boschi-Pinto C, Lopez AD, Murray CJL, Lozano R, Inoue M, editors. AGE STANDARDIZATION OF RATES: A NEW WHO STANDARD2000.

[51] StataCorp. Stata Statistical Software. Release 17. College Station, TX: StataCorp LLC; 2021.

[52] StataCorp. Stata 17 Base Reference Manual College Station, TX: Stata Press; 2021 [cited 2021]. Available from: https://www.stata.com/manuals/r.pdf.

[53] Nyaga VN, Arbyn M, Aerts M. Metaprop: a Stata command to perform meta-analysis of binomial data. Arch Public Health. 2014;72(1):39. 10.1186/2049-3258-72-39 PMC4373114 25810908

[54] Hughes R, Lawrence M. Review Article Globalisation, food and health in Pacific Island countries. Asia Pac J Clin Nutr. 2005;4:298–306. 16326635

[55] Di Tecco C, Fontana L, Adamo G, Petyx M, Iavicoli S. Gender differences and occupational factors for the risk of obesity in the Italian working population. BMC Public Health. 2020;20(1):706. 10.1186/s12889-020-08817-z PMC7229582 32416721

[56] Ng M, Fleming T, Robinson M, Thomson B, Graetz N, Margono C, et al. Global, regional, and national prevalence of overweight and obesity in children and adults during 1980-2013: a systematic analysis for the Global Burden of Disease Study 2013. Lancet. 2014;384(9945):766–81. 10.1016/S0140-6736(14)60460-8 PMC4624264 24880830

[57] Kanter R, Caballero B. Global gender disparities in obesity: a review. Adv Nutr. 2012;3(4):491–8. 10.3945/an.112.002063 PMC3649717 22797984

[58] Centres for Disease Control and Prevention (CDC). Adult Obesity Facts US: CDC; 2021 [cited 2022 January 15]. Available from: https://www.cdc.gov/obesity/data/adult.html.

[59] Rush E, Plank L, Laulu M, Robinson S. Prediction of percentage body fat from anthropometric measurements: Comparison of New Zealand European and Polynesian young women. The American journal of clinical nutrition. 1997;66:2–7. 10.1093/ajcn/66.1.2 9209162

[60] World Bank. Samoan Kids ‘Eat a Rainbow’ for Healthier Lives Washington: IBRD; 2018 [cited 2022 January]. Available from: https://www.worldbank.org/en/news/feature/2018/04/23/samoan-kids-eat-a-rainbow-for-healthier-lives.

[61] Pacific Island Productions. Pacific Island Food Revolution, New Zealand [cited 2022 February]. Available from: https://www.pacificislandfoodrevolution.com/.

[62] PHAMA. The Pacific Horticultural and Agricultural Market Access Program (PHAMA) 2022. Available from: https://phamaplus.com.au/.

[63] Lim S, al e. A comparative risk assessment of burden of disease and injury attributable to 67 risk factors and risk factor clusters in 21 regions, 1990–2010: a systematic analysis for the Global Burden of Disease Study 2010. Lancet. 2012;380(9859). 10.1016/S0140-6736(12)61766-8 PMC4156511 23245609

[64] World Health Organization. Increasing fruit and vegetable consumption to reduce the risk of noncommunicable diseases Biological, behavioural and contextual rationale Geneva2014 [cited 2022]. Available from: https://www.who.int/elena/titles/bbc/fruit_vegetables_ncds/en/.

[65] Frank SM, Webster J, McKenzie B, Geldsetzer P, Manne-Goehler J, Andall-Brereton G, et al. Consumption of Fruits and Vegetables Among Individuals 15 Years and Older in 28 Low- and Middle-Income Countries. J Nutr. 2019;149(7):1252–9. 10.1093/jn/nxz040 31152660

[66] Bell C, Latu C, Coriakula J, Waqa G, Snowdon W, Moodie M. Fruit and vegetable import duty reduction in Fiji to prevent obesity and non-communicable diseases: a case study. Public Health Nutr. 2020;23(1):181–8. 10.1017/S1368980019002660 PMC10200428 31547897

[67] Olney D, Singh R, Schreinemachers P, Hodur J. “Eat more fruit and vegetables” [Internet]. International Food Policy Research Institute, editor2022. [cited 2022]. Available from: https://www.ifpri.org/blog/improving-fruit-and-vegetable-consumption-will-require-holistic-approach.

[68] Malik VS, Hu FB. The role of sugar-sweetened beverages in the global epidemics of obesity and chronic diseases. Nat Rev Endocrinol. 2022;18(4):205–18. 10.1038/s41574-021-00627-6 PMC8778490 35064240

[69] Lo VYT, Sacks G, Gearon E, Bell C. Did imports of sweetened beverages to Pacific Island countries increase between 2000 and 2015? BMC Nutr. 2021;7(1):13. 10.1186/s40795-021-00416-4 PMC8135168 34011416

[70] Reeve E, Thow AM, Namohunu S, Bell C, Lal A, Sacks G. Action-oriented prospective policy analysis to inform the adoption of a fiscal policy to reduce diet-related disease in the Solomon Islands. Health Policy and Planning. 2021. 10.1093/heapol/czab031 PMC8428604 33826719

[71] Kessaram T, McKenzie J, Girin N, Merilles OE, Jr., Pullar J, Roth A, et al. Overweight, obesity, physical activity and sugar-sweetened beverage consumption in adolescents of Pacific islands: results from the Global School-Based Student Health Survey and the Youth Risk Behavior Surveillance System. BMC Obes. 2015;2:34. 10.1186/s40608-015-0062-4 PMC4572651 26401344

[72] Rocha LL, Pessoa MC, Gratao LHA, do Carmo AS, Cordeiro NG, Cunha CF, et al. Characteristics of the School Food Environment Affect the Consumption of Sugar-Sweetened Beverages Among Adolescents. Front Nutr. 2021;8:742744. 10.3389/fnut.2021.742744 PMC8531082 34692751

[73] Australian Institute of Health and Welfare. High blood pressure: Web report. Canberra, ACT: AIHW, 2021.

[74] LaMonica LC, McGarvey ST, Rivara AC, Sweetman CA, Naseri T, Reupena MS, et al. Cascades of diabetes and hypertension care in Samoa: Identifying gaps in the diagnosis, treatment, and control continuum – a cross-sectional study. Lancet Reg Health West Pac. 2022;18. 10.1016/j.lanwpc.2021.100313 PMC8669362 35024652

[75] World Health Organization. Hypertension Factsheet. Geneva: World Health Organization; 2021. Available from: https://www.who.int/news-room/fact-sheets/detail/hypertension. [cited 2022 January 15]

[76] Anderson I. The Economic Costs of Noncommunicable Diseases in the Pacific Islands- A Rapid Stocktake of the situation in Samoa, Tonga and Vanuatu. The World Bank; 2012.

[77] Schwingshackl L, Bogensberger B, Bencic A, Knuppel S, Boeing H, Hoffmann G. Effects of oils and solid fats on blood lipids: a systematic review and network meta-analysis. J Lipid Res. 2018;59(9):1771-82. 10.1194/jlr.P085522 PMC6121943 30006369

[78] Sun Y, Neelakantan N, Wu Y, Lote-Oke R, Pan A, van Dam RM. Palm Oil Consumption Increases LDL Cholesterol Compared with Vegetable Oils Low in Saturated Fat in a Meta-Analysis of Clinical Trials. J Nutr. 2015;145(7):1549-58. 10.3945/jn.115.210575 25995283

[79] Baylin A, Deka R, Tuitele J, Viali S, Weeks DE, McGarvey ST. INSIG2 variants, dietary patterns and metabolic risk in Samoa. Eur J Clin Nutr. 2013;67(1):101-7. 10.1038/ejcn.2012.124 PMC3634362 22968099

[80] Shahid M, Waqa G, Pillay A, Kama A, Tukana IN, McKenzie BL, et al. Packaged food supply in Fiji: nutrient levels, compliance with sodium targets and adherence to labelling regulations. Public Health Nutr. 2021;24(13):4358-68. 10.1017/S136898002100224X PMC10195241 34008486

[81] Thow AM, Priyadarshi S. Aid for Trade: an opportunity to increase fruit and vegetable supply. Bull World Health Organ. 2013;91(1):57-63. 10.2471/BLT.12.106955 PMC3537247 23397351

[82] Thow AM. Trade liberalisation and the nutrition transition: mapping the pathways for public health nutritionists. Public Health Nutr. 2009;12(11):2150-8. 10.1017/S1368980009005680 19433005

[83] Snowdon W, Thow AM. Trade policy and obesity prevention: challenges and innovation in the Pacific Islands. Obes Rev. 2013;14 Suppl 2:150-8. 10.1111/obr.12090 24102909

[84] Sobers N, Samuels TA. Diet and childhood obesity in small island developing states. Lancet Child Adolesc Health. 2019;3(7):445-7. 10.1016/S2352-4642(19)30149-X 31178025

[85] Astika R, Snowdon W, Drauna AM. Exposure to advertising of 'junk food' in Fiji. 2013.

[86] Mialon M, Swinburn B, Wate J, Tukana I, Sacks G. Analysis of the corporate political activity of major food industry actors in Fiji. Glob Health. 2016;12(1):18. 10.1186/s12992-016-0158-8 PMC4862126 27160250

[87] World Health Organization. The updated Appendix 3 of the WHO Global NCD Action Plan 2013-2020. Geneva: World Health Organization, 2018.

[88] Ravuvu A, Waqa G. Childhood Obesity in the Pacific: Challenges and Opportunities. Curr Obes Rep. 2020;9(4):462-9. 10.1007/s13679-020-00404-y 33079338

[89] Reeve E, Thow AM, Bell C, Engelhardt K, Gamolo-Naliponguit EC, Go JJ, et al. Implementation lessons for school food policies and marketing restrictions in the Philippines: a qualitative policy analysis. Glob Health. 2018;14:1-N.PAG. 10.1186/s12992-017-0320-y PMC5781266 29361951

[90] Pacific Islands Forum Secretariat, Secretariate of Pacific Communities. Joint Forum Economic and Pacific Health Ministers Meeting Outcomes Statement. Honiara: 2014.

[91] Snowdon W, Lawrence M, Schultz J, Vivili P, Swinburn B. Evidence-informed process to identify policies that will promote a healthy food environment in the Pacific Islands. Public Health Nutr. 2010;13(6):886-92. 10.1017/S136898001000011X 20196907

[92] McKenzie BL, Santos JA, Geldsetzer P, Davies J, Manne-Goehler J, Gurung MS, et al. Evaluation of sex differences in dietary behaviours and their relationship with cardiovascular risk factors: a cross-sectional study of nationally representative surveys in seven low- and middle-income countries. Nutr J. 2020;19(1):3. 10.1186/s12937-019-0517-4 PMC6956488 31928531

[93] Flood D, Guwatudde D, Damasceno A, Manne-Goehler J, Davies JI. Maximising use of population data on cardiometabolic diseases. Lancet Diabetes Endocrinol. 2022. 10.1016/S2213-8587(21)00328-4 PMC10072128 35026159

