# Supplementary material for: The tide of dietary risks for noncommunicable diseases in Pacific Islands: an analysis of population NCD surveys
Source: BMC Public Health. 2022 Aug 10;22:1521. doi: 10.1186/s12889-022-13808-3 (PMC9364577; doi:10.1186/s12889-022-13808-3)
Supplement: Supplementary file 1 — Additional file 1. [file 12889_2022_13808_MOESM1_ESM.docx]

# **The tide of dietary risks for noncommunicable diseases in Pacific Islands: an analysis of population NCD surveys: Supplementary File 1**

**Graphed results**

**Overweight and obesity**

**Figure 1: Age-standardized pooled prevalence of adults living with overweight and obesity for Pacific Island countries (2002-2009)**

**Figure 2: Age-standardized pooled prevalence of adults living with overweight and obesity for Pacific Island countries (2011-2019)**

**Figure 3: Age-standardized pooled prevalence of males living with overweight and obesity for Pacific Island countries (2002-2009)**

**Figure 4: Age-standardized pooled prevalence of males living with overweight and obesity for Pacific Island countries (2011-2019)**

**Figure 5: Age-standardized pooled prevalence of females living with overweight and obesity for Pacific Island countries (2002-2009)**

**Figure 6: Age-standardized pooled prevalence of females living with overweight and obesity for Pacific Island countries (2011-2019)**

**Figure 7: Age-standardized prevalence of adults aged 25-69 years living with overweight and obesity by survey year and Pacific Island Country and Territory**

*Error bars represent confidence interval, number in the bar indicates prevalence for the specific survey.*

**Figure 8: Age-standardized prevalence of females aged 25-69 years living with overweight and obesity by survey year and Pacific Island Country and Territory**

**Figure 9: Age-standardized prevalence of males aged 25-69 years living with overweight and obesity by survey year and Pacific Island Country and Territory**

**Fruit and vegetable consumption**

**Figure 10: Age-standardized pooled prevalence of adults consuming less than five servings of fruits and vegetables per day for Pacific Island countries**

**Figure 11: Age-standardized pooled prevalence of adults consuming less than five servings of fruits and vegetables per day for Pacific Island countries**

**Figure 12: Age-standardized pooled prevalence of males consuming less than five servings of fruits and vegetables per day for Pacific Island countries**

**Figure 13: Age-standardized pooled prevalence of males consuming less than five servings of fruits and vegetables per day for Pacific Island countries**

**Figure 14: Age-standardized pooled prevalence of females consuming less than five servings of fruits and vegetables per day for Pacific Island countries**

**Figure 15: Age-standardized pooled prevalence of females consuming less than five servings of fruits and vegetables per day for Pacific Island countries**

**Figure 16: Age-standardized prevalence of adults aged 25-69 years consuming less than five servings of fruits and vegetables per day by survey year and Pacific Island Country and Territory**

**Figure 17: Age-standardized prevalence of females aged 25-69 years consuming less than five servings of fruits and vegetables per day by survey year and Pacific Island Country and Territory**

**Figure 18: Age-standardized prevalence of males aged 25-69 years consuming less than five servings of fruits and vegetables per day by survey year and Pacific Island Country and Territory**

**Hypertension**

**Figure 19: Age-standardized pooled prevalence of adults living with hypertension (SBP>140 and/or DBP>90 mmHg and/or currently living with medication) for Pacific Island countries (2002-2009)**

**Figure 20: Age-standardized pooled prevalence of adults living with hypertension (SBP>140 and/or DBP>90 mmHg and/or currently living with medication) for Pacific Island countries (2011-2019)**

**Figure 21: Age-standardized pooled prevalence of males living with hypertension (SBP>140 and/or DBP>90 mmHg and/or currently living with medication) for Pacific Island countries (2002-2009)**

**Figure 22: Age-standardized pooled prevalence of males living with hypertension (SBP>140 and/or DBP>90 mmHg and/or currently living with medication) for Pacific Island countries (2011-2019)**

**Figure 23: Age-standardized pooled prevalence of females living with hypertension (SBP>140 and/or DBP>90 mmHg and/or currently living with medication) for Pacific Island countries (2002-2009)**

**Figure 24: Age-standardized pooled prevalence of females living with hypertension (SBP>140 and/or DBP>90 mmHg and/or currently living with medication) for Pacific Island countries (2011-2019)**

**Figure 25: Age-standardized prevalence of adults aged 25-69 years living with hypertension by survey year and Pacific Island Country and Territory**

**Figure 26: Age-standardized prevalence of females aged 25-69 years living with hypertension by survey year and Pacific Island Country and Territory**

**Figure 27: Age-standardized prevalence of males aged 25-69 years living with hypertension by survey year and Pacific Island Country and Territory**

**Diabetes**

**Figure 28: Age-standardized prevalence of adults aged 25-69 years living with diabetes by survey year and Pacific Island Country and Territory**

**Figure 29: Age-standardized prevalence of females aged 25-69 years living with diabetes by survey year and Pacific Island Country and Territory**

**Figure 30: Age-standardized prevalence of males aged 25-69 years living with diabetes by survey year and Pacific Island Country and Territory**

**Hypercholesterolemia**

**Figure 31: Age-standardized pooled prevalence of adults aged 25-69 years living with raised total cholesterol, round 1**

**Figure 32: Age-standardized pooled prevalence of adults aged 25-69 years living with raised total cholesterol, round 2**

**Figure 33: Age-standardized pooled prevalence of males aged 25-69 years living with raised total cholesterol, round 1**

**Figure 34: Age-standardized pooled prevalence of males aged 25-69 years living with raised total cholesterol, round 2**

**Figure 35: Age-standardized pooled prevalence of females aged 25-69 years living with raised total cholesterol, round 1**

**Figure 36: Age-standardized pooled prevalence of females aged 25-69 years living with raised total cholesterol, round 2**

**Figure 37: Age-standardized prevalence of adults aged 25-69 years living with raised total cholesterol by survey year and Pacific Island Country and Territory**

**Figure 38: Age-standardized prevalence of females aged 25-69 years living with raised total cholesterol by survey year and Pacific Island Country and Territory**

**Figure 39: Age-standardized prevalence of males aged 25-69 years living with raised total cholesterol by survey year and Pacific Island Country and Territory**
